# Challenging the spliceosome machine

**DOI:** 10.1186/gb-2006-7-1-r3

**Published:** 2006-01-17

**Authors:** Michael Weir, Matthew Eaton, Michael Rice

**Affiliations:** 1 Department of Biology, Wesleyan University, Middletown, CT 06459, USA; 2 Department of Mathematics and Computer Science, Wesleyan University, Middletown, CT 06459, USA

## Abstract

**Background:**

Using cDNA copies of transcripts and corresponding genomic sequences from the Berkeley Drosophila Genome Project, a set of 24,753 donor and acceptor splice sites were computed with a scanning algorithm that tested for single nucleotide insertion, deletion and substitution polymorphisms. Using this dataset, we developed a progressive partitioning approach to examining the effects of challenging the spliceosome system.

**Results:**

Our analysis shows that information content increases near splice sites flanking progressively longer introns and exons, suggesting that longer splice elements require stronger binding of spliceosome components. Information also increases at splice sites near very short introns and exons, suggesting that short splice elements have crowding problems. We observe that the information found at individual splice sites depends upon a balance of splice element lengths in the vicinity, including both flanking and non-adjacent introns and exons.

**Conclusion:**

These results suggest an interdependence of multiple splicing events along the pre-mRNA, which may have implications for how the macromolecular spliceosome machine processes sets of neighboring splice sites.

## Abstract 2

Analysis of a set of almost 25,000 donor and acceptor splice sites in *Drosophila* shows that information content increases near splice sites flanking very long of very short introns and exons.

## Background

The genomic era has heralded the availability of vast quantities of sequence data that has raised the need for effective conceptual frameworks for analyzing sequences on a large scale. The concept of information [1-3] provides a powerful quantitative measure of sequence conservation, allowing functional properties of sequences to be derived through multiple analytical approaches. Specifically, the information at each nucleotide position *p *for a set of *n *aligned sequences is defined by the expression:

*information*(*p*) = 2 - Σ{-*f*_p_(α) *log*_2_(*f*_p_(α)) | α = A, C, G, or U} - γ

The summation represents the uncertainty based on the frequencies of occurrence *f*_p_(A), ..., *f*_p_(U) of the nucleotides A, ..., U at position *p*. The sampling correction factor γ depends on *n *and decreases toward 0 as the value of *n *increases [2,4]. In general, the information at each nucleotide position lies on a continuous scale between 0 bits (random sequence) and 2 bits (exactly one conserved base at that position). The cumulative or total information for a set of aligned sequences of length *m *is defined by the expression:

*information*(1..*m*) = Σ{*information*(*p*) | 1 ≤ *p *≤ *m*}

By comparing sets of sequences that reflect different degrees of 'strain' on a biological machine, it is possible to gain important insights into relationships within the biological system. For example, by comparing subsets of *Drosophila *splice sites next to progressively longer introns, we observed progressively larger amounts of information at the sites, reflecting a need for stronger binding sites with longer introns [4]. This 'progressive partitioning' approach can uncover subtle trends that are statistically significant. In this study, we have extended the powerful approach to examine a set of 32,987 exons and have discovered that stronger sequence conservation is also associated with longer exons, as observed previously with introns. But we have also observed a new result, namely that there is enhanced sequence conservation for very short exons and introns, suggesting that the spliceosome machine is also strained by very short splice elements.

Although the trends observed in progressive partitioning analyses, such as those described above, reflect properties of groups of sequences, there will generally exist some sequences within the group that do not conform well to the trends. By focusing on these 'non-conformers', it is possible to identify properties that compensate for the poor match to the trend. For example, using a 'forced mismatch' approach to identify non-conformers, we found previously that splice sites with poor matches to the common nucleotide choices adjacent to the splice sites (sometimes described as a 'consensus sequence') instead have compensating enrichment in A nucleotide content near the splice site. This enrichment in A content may facilitate spliceosome function by reducing the likelihood of RNA secondary structure [5]. The forced mismatch approach we described previously compared sets of sequences with small numbers of matches to conserved nucleotides at positions near the splice site (for example, 5 of 7 matches at donor positions -1 to +6, abbreviated D-1 to D6) to sets with many matches (for example, 7 of 7). Unfortunately, this analytical approach assigns equal weighting to each of the conserved nucleotide positions, regardless of how strong the conservation is at each position. Instead, it would be better to score sequences in a way that takes into account the degree of conservation at each nucleotide position such that mismatches at highly conserved nucleotide positions are treated as more important than those at less conserved positions. Indeed, this problem highlights a more general need in molecular biology to be able to score individual instances of conserved motifs so that their functions can be assessed quantitatively.

This problem can be overcome by using an information measure for individual sequences described in [6]. This measure assigns greater weight to nucleotide positions that are more highly conserved. The basic idea is the following: suppose that our reference set *S *consists of *n *aligned sequences, each of length *m*, and *s*_1_,..., *s*_m _denotes the nucleotides in a sequence *s *∈ *S*. Then the individual information of *s *is defined by:

*score*(*s*) = Σ{2 + *log*_2_(*f*_p_(*s*_p_)) - γ | 1 ≤ *p *≤ *m*}

where *f*_p_(*s*_p_) denotes the frequency of occurrence of nucleotide *s*_p _at position *p *and γ denotes the sampling correction factor discussed above. This score is a real number that provides a quantitative assessment of how well *s *conforms to the conservation determined by the alignment. The set of individual information scores for a set of sequences defines a distribution that has the average value *information*(1..*m*).

Ignoring the correction γ, the contribution to the score at a nucleotide position approaches +2 if the nucleotide present is almost completely conserved at that position. The contribution is 0 if the nucleotide present normally occurs with probability 0.25, and is negative (potentially significantly smaller than 0) if the nucleotide present occurs very infrequently at that position. Hence, the value of the individual information *score*(*s*) is at most 2*m*.

In some cases, we want to assess how well a nucleotide sequence *s *conforms to the consensus represented by *S *even if it is not a member. To define a score for *s*, which may contain at some positions nucleotides not found in the original alignment, we replace the frequencies *f*_p_(α) with frequencies based on pseudocounts. These counts are based on the assumption that each nucleotide potentially occurs at least once at every position (see Materials and methods). Then the individual information of *s *is defined as above.

The distributions of individual information scores provided the basis for the forced mismatch and progressive partitioning analyses described below. These analyses, as well as measurements of cumulative information and nucleotide content over broad regions near splice sites, permitted us to strain the spliceosome machine and thereby gain insights into how sets of pre-mRNA splice sites are processed. As discussed previously [4,7], the studies described below harness the strengths of relational databases as frameworks for the analysis of large genomic datasets. Indeed, our *Drosophila *splice site database is indispensable for carrying out the work described in this paper.

## Results and discussion

### Sequence mismatches and polymorphisms

We previously analyzed a set of 10,057 introns in 3,090 cDNAs [4]; 514 additional cDNAs were predicted to have no introns. Taking advantage of a larger set of 10,284 cDNA sequences posted at the Berkeley Drosophila Genome Project (BDGP), we used BLAST to identify corresponding genomic sequences for 10,057 of these cDNAs. Using an improved scanning algorithm for computing splice sites, we identified 24,753 introns in 7,062 of these cDNAs; 1,172 additional cDNAs had no introns and the scanning algorithm failed for the remaining 1,823 cDNAs, which were not included in our dataset (Table 1). The new algorithm (described in Materials and methods, and Additional data file 1) permitted limited sequence mismatches or polymorphisms between the cDNA and corresponding genomic sequences - single nucleotide substitutions and single nucleotide deletions or insertions. Sequence mismatches were due in part to the lower sequence quality of the reverse-transcriptase-derived cDNAs (>97% accurate) compared to the high-quality genomic sequences (1 error in 100,000 nucleotides) [8,9]. The genomic nucleotide sequences surrounding the predicted splice sites were stored in a relational database as described previously [4,7]. The database can be accessed at [10].

Allowing for single-nucleotide mismatches (substitutions and gaps) increased substantially the number of cDNAs successfully parsed by our scanning algorithm - from 5,092 to 8,234 (Table 1). We assessed the quality of the predicted splice sites by examining conformity to the canonical consensus GU... AG or secondary consensus AU...AC at positions D1, D2 and A-2, A-1 [11-14]. We observed previously [4] that predicted introns or exons of length <20 nucleotides were poor quality based on their reduced adherence to the canonical consensus. The new scanning algorithm predicted far fewer splice elements <20 nucleotides (0.14% of 57,740; Table 1) compared to our previous algorithm, but these had almost as low adherence to the canonical consensus as observed previously. Disregarding the 75 cDNAs in our new dataset with splice elements of length <20 nucleotides, 99.1% of the predicted introns conformed to the consensus GU...AG or AU...AC at the four canonical positions. Of these 24,193 introns, 7 had the secondary consensus AU...AC. This compares favorably with our previous smaller dataset in which 99.2% of introns in cDNAs with splice elements of length ≥20 conformed to the consensus at the four canonical positions. In the analysis described below, we restricted our attention to the new dataset consisting of 8,159 cDNAs with splice elements of length ≥20 (Additional data file 3).

The 8,159 cDNAs represent mRNAs from 7,268 different genes. Of these, 768 of the genes have two or more cDNAs, and the cDNAs for 378 of these genes exhibit alternative splicing in our dataset. However, future expansion of the cDNA dataset will likely reveal alternative splicing in a much larger fraction of the genes.

### Correlating splice element lengths with information

**Figure 1:**
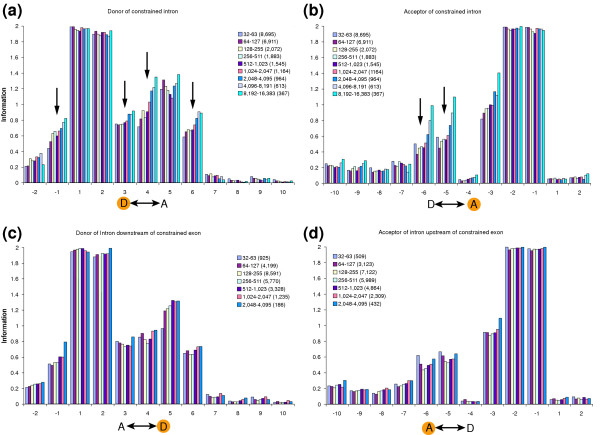
Information varies with intron and exon length. Donor and acceptor sites flanking either long or very short introns or exons have increased information. **(a,b) **The graphs show information profiles for nucleotide positions near donor and acceptor sites for nine sets of introns corresponding to progressively larger length ranges. We calculated the standard deviation at each of nucleotide positions D-2 to D10 and A-10 to A2. The maximum standard deviation observed was 0.073 bits (see [4] for explanation of standard deviation calculations). **(c,d) **Equivalent graphs based on varying exon length. The maximum standard deviation at each nucleotide position is 0.040 bits, except for the 2,048 to 4,095 size class where the value is 0.104 bits. Arrows mark nucleotide positions with characteristic information profile trends. Orange circles show which splice site is graphed relative to the varied intron or exon (double arrow).

**Figure 2:**
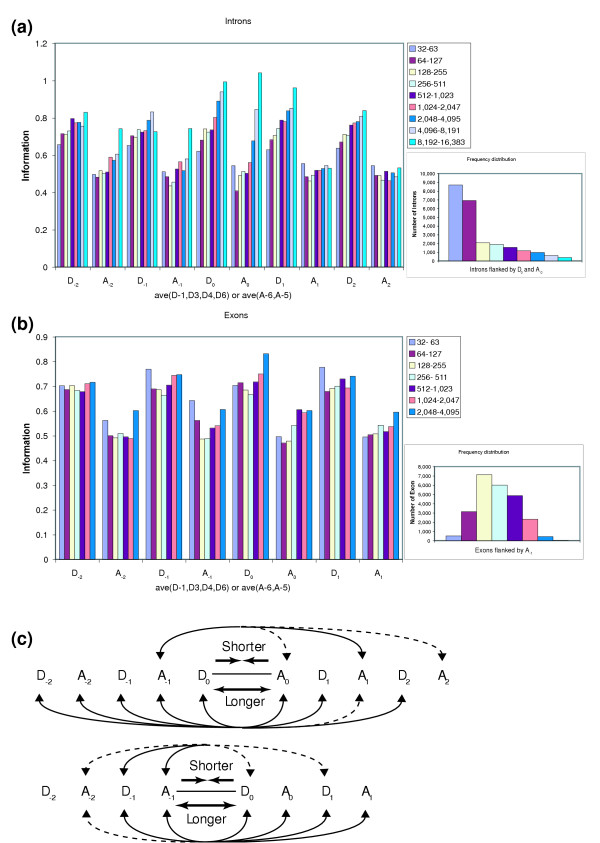
Neighborhood effects on splice site strength. Nucleotide positions D-1, D3, D4, D6, A-6 and A-5 show pronounced changes in information levels when intron or exon lengths are varied (see Figure 1). The figure illustrates the effects at these nucleotide positions of donor and acceptor sites in the neighborhood. The subscript labeling specifies how far the donor or acceptor sites are from the introns or exons being varied, as defined below. **(a,b) **The average information levels (ave(D-1, D3, D4, D6) or ave(A-6, A-5)) are plotted for **(a) **nine intron length or **(b) **seven exon length ranges. (a) The varied introns are flanked by donor D_0 _and acceptor A_0_. **(b) **The varied exons are flanked by acceptor A_-1 _and donor D_0_. Length frequency distributions are shown for **(a) **the introns flanked by D_0 _and A_0 _and **(b) **the exons flanked by A_-1_. **(c) **The figure illustrates the donor and acceptor sites in the neighborhood whose adjacent nucleotide positions showed elevated information with shorter introns or exons (upper arrows) or longer introns or exons (lower arrows). Solid arrows depict strong effects; dashed arrows show weak effects.

We showed previously that donor and acceptor sites near long introns have higher levels of information when compared to short splice elements [4]. Our new larger dataset confirms this result: information levels increase with progressively longer intron length ranges. This observation applies to splice sites immediately flanking the varied intron (Figure 1a,b), as well as more distant splice sites (Figure 2a,c). Indeed, significant progressive increases in information are observed at positions D-1, D3, D4, D6, A-6 and A-5 (arrows in Figure 1a,b), and these nucleotide positions also show increases in information at splice sites not flanking the varied intron (Figure 2a).

In this study, we also examined the effects of increasing exon length. As with introns, increased exon lengths are also associated with increases in information at donor and acceptor sites, although the effects are a little less pronounced, especially for donor sites (Figure 1c,d). Unlike our previous observations for introns, however, we found that information also increases for shorter exons, particularly at positions A-5, A-6, D3 and D4, the same nucleotide positions with particularly enhanced information values for longer introns (Figure 1c,d). This observation suggests that the spliceosome machinery is strained by both longer and shorter exons, and is least strained for exons of intermediate length. Very short exons may cause crowding problems for RNA-binding molecules, leading to a need for stronger donor and acceptor sites flanking the exon. Indeed, previous small scale studies have suggested that very short exons can be detrimental to splicing [15,16], and that increasing splice site strength can alleviate this problem [17]. Previous observations [18,19] have also suggested that long exons can be detrimental to splicing, although other studies [20] have questioned the general applicability of this hypothesis [3]. We found that the information levels at some non-flanking splice sites also increased for both very long and very short exons, as summarized in Figure 2b,c. This observation suggests that strain caused by either very long or very short exons can be counterbalanced by having stronger spliceosome binding sites at splice sites in the neighborhood.

Given these observations for short exons, we also extended our analysis of introns to include shorter intron length ranges than examined previously. This analysis revealed subtle increases in information at some donor and acceptor sites in the neighborhood of very short introns when compared to slightly longer introns (Figures 1b and 2a,c). We conclude that for both introns and exons, short splice element length strains the spliceosome machine when compared to elements of intermediate length.

These results predict that there would be selective pressure for exons and introns of intermediate length, and against shorter or longer splice elements. This assertion is consistent with the observed length distributions of the splice elements because the median intron and exon lengths lie in length classes with smaller information values near the left-end of the information curves (Figure 2a,b). This model is further supported by observations that splice site mutations often uncover the use of cryptic splice sites that are very close to the mutated site but are not normally used [18,19], again indicating a preference for intermediate splice element length by the spliceosome machinery. Moreover, the artificial lengthening of exons can similarly reveal cryptic sites in the exon [18,19].

It has been suggested (TD Schneider, personal communication; RK Shutzaberger, L Smith, I Lyakhov, R Fisher, TD Schneider, in preparation) that higher information at splice sites is associated with decreased off rates for spliceosome-pre-mRNA molecular interactions. According to this hypothesis, our results suggest that when the spliceosome processes pre-mRNAs with either very long or very short splice elements, it is advantageous to increase the stability (reduce off rates) of the spliceosome-pre-mRNA interactions. Increased stability could be particularly useful to counteract molecular crowding problems near small introns and exons.

### Cumulative information

**Figure 3:**
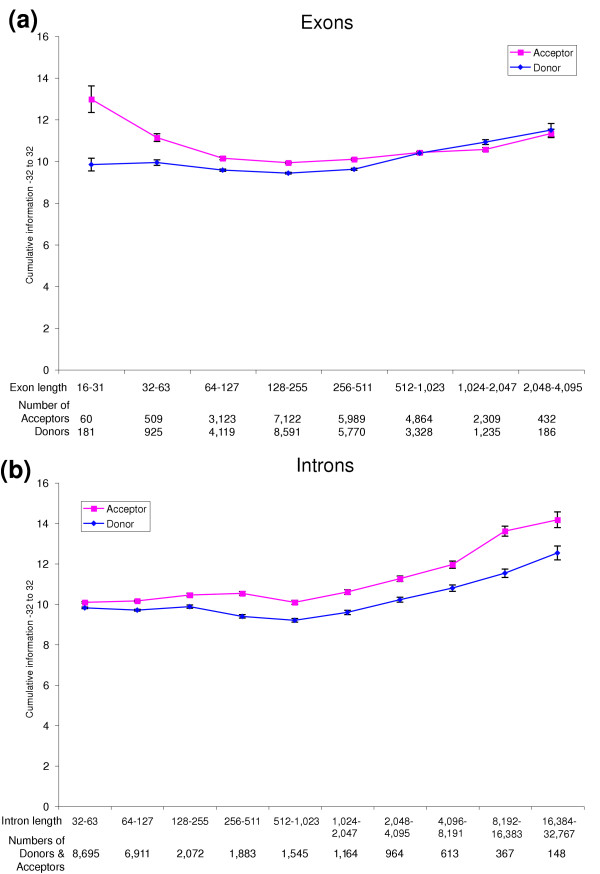
Cumulative information. Cumulative information for positions -32 to +32 of donor and acceptor sites is plotted for contiguous **(a) **exon or **(b) **intron length ranges. Error bars show standard deviations.

The analysis described above examined trends in information content at individual nucleotide positions of aligned sets of sequences. It is also useful to examine the cumulative information over adjacent nucleotide positions. For example, the cumulative information measured from positions -32 to +32 of donor or acceptor sites increases progressively for longer exon length ranges (Figure 3a). Cumulative information also increases significantly for shorter exons compared to exons of intermediate length (Figure 3a), confirming our observations at individual nucleotide positions (Figure 1c,d). The same trends are observed for longer introns (Figure 3b). However, shorter introns do not show significantly elevated cumulative information (but see regional nucleotide content analysis below).

From theoretical considerations [2], there is a minimum sufficient amount of information required to uniquely specify sites with a given average spacing in random sequence. For example, a six-cutter restriction enzyme cuts every 4^6 ^(= 2^12^) bases on average and the aligned restriction sites have 12 bits of information. In general, donor and acceptor sites have 9 to 13 bits and 10 to 16 bits of information, respectively, depending upon the lengths of adjacent splice elements. These cumulative information values suggest that there could be sufficient information to specify the splice sites in the observed splice element length ranges. Several authors have discussed this point of view [2,13]. However, this general view relating sequence information content to the expected frequencies of splice sites assumes that the recognition of splice sites on a pre-mRNA are independent events, and the view does not take into consideration possible constraints imposed by the spliceosome machine. Indeed, the interrelationships between neighboring splice sites discussed above (Figure 2c), and further elaborated below, suggest that the recognition of splice sites are not independent events. This indicates that cumulative information measurements at individual splice sites are not good indicators of expected frequencies of splice sites.

### Regional nucleotide content

To assess further the nucleotides at positions -32 to +32 of splice sites, we carried out a statistical analysis of nucleotide content in these regions. In our previous work, we compared sets of splice sites near long introns and near short introns [4]. In addition to the position-specific effects, as described above, we observed characteristic changes in regional nucleotide content. For example, we found characteristic increases in C and U content in the pyrimidine tracks upstream of the acceptor of the intron whose length was being varied, whereas the increase was more pronounced for U in the acceptor of the upstream intron, and for C in the downstream acceptor (these acceptors are labeled A_-1 _and A_1_, respectively, in Figure 2).

**Figure 4:**
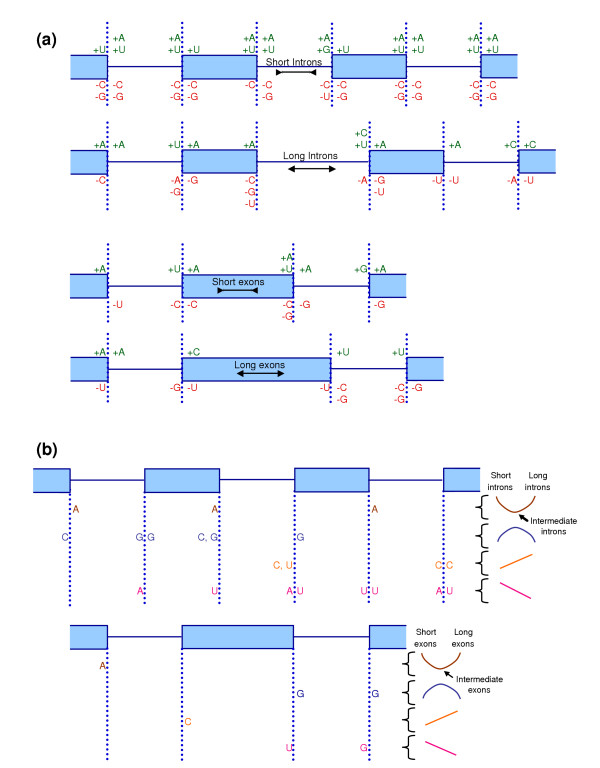
Regional nucleotide content near splice sites. Differences in regional nucleotide content measured in 32 nucleotide regions adjacent to splice sites in the neighborhood of a varied intron or exon. Filled in rectangles denote exons; solid lines denote introns. **(a) **The comparisons made were: short introns (48 to 59 nucleotides (nt); *n *≥ 3,417) with intermediate introns (64 to 1,023 nt; *n *≥ 8953); long introns (2,048 to 16,383 nt; *n *≥ 1,070) with intermediate introns; short exons (32 to 90 nt; *n *≥ 1,515) with intermediate exons (128 to 511 nt; *n *≥ 13,274); long exons (1,048 to 4,095 nt; *n *≥ 1,364) with intermediate exons; where *n *denotes the sample size of each group. In each region, nucleotide contents were compared using a bootstrap alternative to the two-sample *t* test at the 1% significance level (see Materials and methods). Compared to intermediate introns or exons, short or long splice elements with significantly higher (or lower) nucleotide content are illustrated in green: +A, +C, +G, +U (or red: -A, -C, -G, -U). **(b) **The nucleotides pictured show significant changes in the indicated region for both short and long introns (or exons) when compared to intermediate length introns (or exons). In some cases, A is enriched, or C or G is depleted for both long and short splice elements. In other cases, A or U (and in one case G) is enriched for short and depleted for long splice elements. There are also cases where C (and in one case U) is depleted for short and enriched for long splice elements.

Given our new observation that very short exons or introns also strain the spliceosome machine, we extended the preceding analysis by using our new larger dataset to compare long or short splice elements to intermediate length elements. Specifically, we used the bootstrap alternative to the two-sample *t* test (see Materials and methods) to compare nucleotide contents in 32 nucleotide long windows adjacent to different groups of splice sites. The bootstrap method allowed us to determine whether observed regional changes in nucleotide levels were significant (Figure 4a,b; Additional data file 2). The percentage changes in nucleotide levels that were significant (*p* < 0.01) were between 0.31% and 3.36% with a mean of 1.63 ± 0.73%.

Based on these tests, we conclude that splicing of short introns as well as short exons appears to be facilitated by increased A and U content and reduced G and C content, perhaps because this lowers the likelihood of RNA secondary structures thereby facilitating spliceosome function. In several 32 nucleotide regions, the same change (A enrichment, or C or G depletion) is observed for both short and long introns (or exons) when compared to intermediate-length elements (Figure 4b). In other cases, the same nucleotide shows opposite effects for short and long elements (Figure 4b). Short introns are associated with purine (A, G) enrichment upstream of their acceptor sites, consistent with previous observations that small introns often lack a pyrimidine track [21,22]. Hence, although the diagnostic acceptor positions -6 and -5 have higher information with smaller introns (Figures 1b and 2a), because the pyrimidine tracts are diminished, the overall cumulative information is not elevated with smaller introns (Figure 3b). This separation of effects suggests that the pyrimidine tract may be involved in different spliceosome molecular interactions than A-6 and A-5, which our progressive partitioning analysis implicates in spliceosome function.

### Individual information

So far, we have discussed the cumulative information measured using groups of aligned sequences. Information can also be defined for individual sequences (see Background). For a given sequence in a set of aligned sequences, each position can be evaluated based on the frequency of occurrence of the given nucleotide in the alignment. Measuring the individual information of a sequence [6] places higher weights on the nucleotide positions with greater conservation (see Materials and methods). A highly conserved nucleotide in the sequence contributes a positive value to the individual information score, and the presence of a very rare nucleotide contributes a significant negative value.

**Figure 5:**
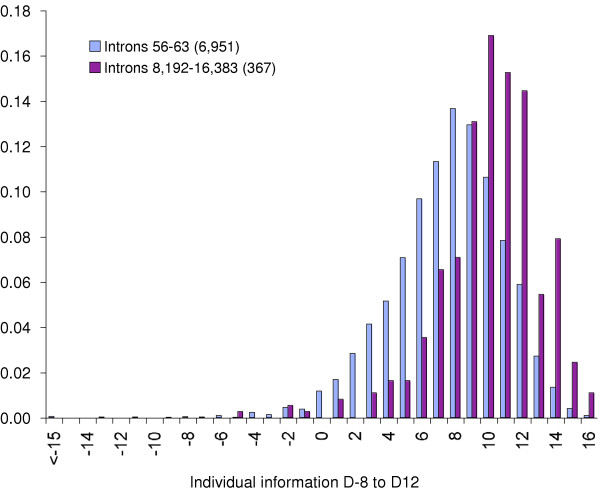
Individual information distributions are sensitive to intron length. Individual information was computed at nucleotide positions -8 to +12 of donor sites flanking introns with lengths 56 to 63 (blue) or 8,192 to 16,383 (red) based on a reference set consisting of introns with lengths 8,192 to 16,383. The mean of the distribution of scores for introns 8,192 to 16,383 (10.01 ± 3.00; *n *= 367) is significantly higher than for introns 56 to 63 (7.57 ± 3.36; *n *= 6951) (*p *< 0.01 by one-tailed *t* test).

We used the donor splice sites (positions D-8 to D12) adjacent to long introns (length 8,192 to 16,383) as a reference set to compute the distributions of individual information scores for several other sets of donor splice sites. For example, the individual information distribution for all donor sites (*n *= 24,423) has a mean of 8.03 ± 3.42 (not shown). As might be expected, if we compare the individual information scores for donor sites adjacent to long introns (lengths 8,192 to 16,383, *n *= 367) with those for short introns (lengths 56 to 63, *n *= 6,951), the mean of the distribution shifts to 10.01 ± 3.00 for the longer introns, and 7.57 ± 3.36 for the shorter introns (Figure 5), consistent with our observation that donor sites flanking longer introns require higher information.

**Figure 6:**
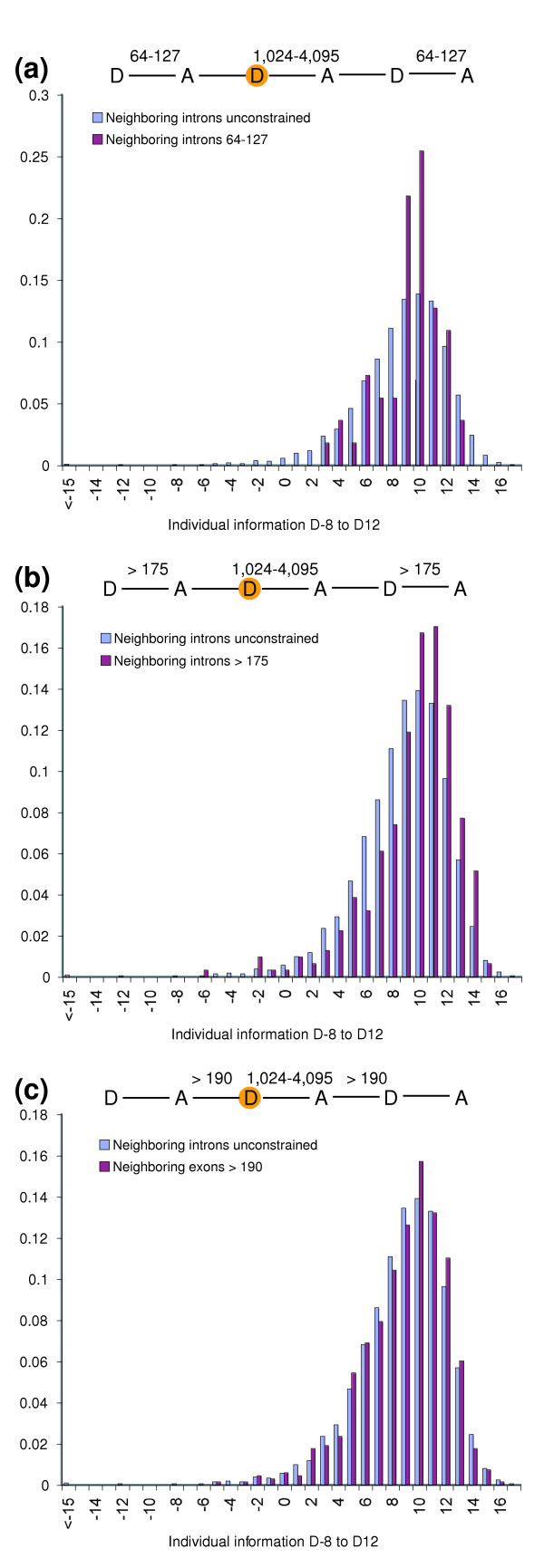
Individual information spread is sensitive to neighborhood constraints. Individual information was computed at nucleotide positions -8 to +12 of donor sites flanking introns with lengths 1,024 to 4,095 based on a reference set of introns with lengths 8,192 to 16,383. For each computation, the neighborhood introns or exons were either constrained (red) or not constrained (blue) as illustrated in the figure. The various datasets used were as follows. **(a) **Introns with lengths 1,024 to 4,095 flanked by introns with lengths 64 to 127 (red); mean individual information = 9.18 ± 2.30 (*n *= 55). **(b) **Introns with lengths 1,024 to 4,095 flanked by introns with lengths >175 (red); mean individual information = 9.56 ± 3.17 (*n *= 311). **(c) **Introns with lengths 1,024 to 4,095 flanked by exons with lengths >190 (red); mean individual information = 8.91 ± 3.07 (*n *= 681). (For comparison, the mean individual information of all introns with lengths 1,024 to 4,095 (blue) is 8.75 ± 3.35 (*n *= 2,128).)

If we further restrict the lengths of neighboring non-flanking introns or exons near the donor site being monitored, we find that the distribution of individual information values is tightened. For example, for introns of length 1,024 to 4,095, restricting the lengths of immediately neighboring introns to 64 to 127 lowers the standard deviation from 3.35 to 2.30 (Figure 6a). In addition, the distribution means are shifted upwards when the lengths of neighboring introns (Figure 6b) or exons (Figure 6c) are increased. The tightening of the distributions suggests that the normal spread is determined, at least in part, by the different lengths of splice elements in the vicinity of the monitored donor sites. This is consistent with and supports the model [4] that the information at splice sites is specified by a balance of forces determined by the lengths of neighboring introns and exons - including both flanking and non-adjacent splice elements (see also Figure 2c). The model suggests that there is interdependence of splicing events along the pre-mRNA. This idea is consistent with experiments in which mutation of donor sites can significantly reduce the removal rate of an upstream intron [23]. A balance between neighboring sites is also suggested by experiments in which deleterious affects of lengthening an exon (causing exon skipping) can be reversed by placing the exon adjacent to shorter introns [20].

This analytical approach, based on examining individual information distributions, provides a useful complement to the more common approach of analyzing information at nucleotide positions in sets of aligned sequences. Unlike the latter approach, the notion of individual information provides insight into the conformity of individual sequences to sequence motifs and is not restricted to the averaged conformity of groups of sequences.

### Forced mismatch

A forced mismatch analysis focuses on subsets of splice sites whose sequences do not conform well to the high-frequency nucleotide choices at the nucleotide positions with high information. Using this technique, previously we uncovered sequence properties that likely facilitate splicing [4]. For example, donor sites with only 5-of-7 matches to the high-frequency nucleotide choices at D-1 to D6 have enhanced A content at neighboring nucleotide positions when compared to donor sites with 7-of-7 matches.

**Figure 7:**
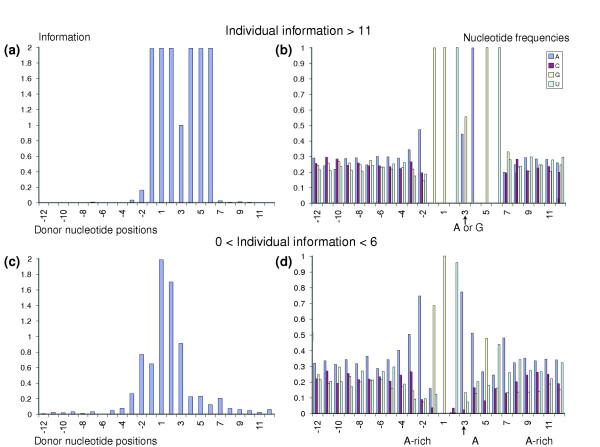
Forced mismatch reveals A enrichment. Individual information was measured at nucleotide positions D-1 to D6 of donor sites flanking introns with lengths 1,024 to 4,095, using a reference set consisting of introns with lengths 8,192 to 16,383. The subset of these donor sites with individual information between 0 and 6 (**(c) **and **(d)**: dataset I) was compared with the subset with individual information > 11 (**(a) **and **(b)**: dataset II). Group information (**(a) **and **(c)**) and nucleotide counts (**(b) **and **(d)**) at each nucleotide position D-12 to D12 are plotted for the two subsets. The average standard deviation in information at nucleotide positions -12 to 12 is 0.030 for dataset I and 0.006 for dataset II. The maximum standard deviation for nucleotide counts at each nucleotide position is 0.026 for dataset I and 0.019 for dataset II. See [4] for a discussion of standard deviation calculations.

In contrast to this approach, the individual information approach described above assigns different weights to different nucleotide positions depending upon the degree of sequence conservation at that position. In principle, this is a superior method for scoring individual sequences. Therefore, we undertook a new forced mismatch analysis based on individual information. We examined subsets of donor sites with suboptimal levels of individual information at D-1 to D6, and compared this subset with the donor sites having high individual information. The analysis was performed on donor sites adjacent to introns with lengths in the range 1,024 to 4,095. The suboptimal donor sites with low individual information values (between 0 and 6) showed enhanced A content on both sides of the D-1 to D6 window when compared to donor sites with high individual information values (>11; Figure 7). Also, analogous to our previous observations, an A nucleotide at D3 was greatly preferred in the suboptimal set when compared to the high-information set that had similar frequencies of A and G at D3 (Figure 7). Therefore, the presence of A at D3 appears to facilitate splicing of suboptimal donor sites. The enhancement of A content in the vicinity of D-1 to D6 may reduce the likelihood of RNA secondary structure and thereby facilitate spliceosome function by increasing the availability of the splice sites for interactions with the spliceosome machinery.

Although our new results using individual information profiles are qualitatively equivalent to the results obtained by our previous un-weighted approach (comparing 5-of-7 to 7-of-7 subsets), the new approach is preferable for future analyses because it provides an appropriate weighting for each nucleotide position in the alignment. As illustrated in Table 2, the donor sites (1,024 to 4,095) with individual information between 0 and 6 have mainly 5-of-7 matches to the consensus GGU[A|G]AGU, although some have 4-of-7 or 6-of-7 matches. For example, the D-1 to D6 sequences GGGAAGU, GGUGACU, GGCAAGU, GGUAACU, and GGUCAGU each have 6-of-7 matches but individual information <6 (3.61, 4.43, 4.61, 5.35, 5.83, respectively). In contrast, the sequences UGUAAGC, AGUAAGA, and AGUAAGC have individual information >6 (6.06, 6.33, 6.46, respectively), but only 5-of-7 matches.

## Conclusion

### Implications for spliceosome function

*Drosophila *splice sites were computed using a scanning algorithm that compared cDNAs with genomic sequences. The efficiency of splice site prediction was enhanced significantly by allowing for sequence-mismatch and polymorphic differences between the cDNA and genomic sequences. A progressive partitioning analysis of our expanded dataset of 24,423 donor and acceptor sites revealed that the spliceosome machine is strained at splice sites near both long introns and long exons. It is also strained at very short splice elements, suggesting that very short elements are associated with crowding problems for the binding of spliceosome components. Our study demonstrates the analytical power of a progressive partitioning analysis of information calculated from sets of aligned sequences. It also shows the analytical versatility of using individual information for scoring sequence conservation near individual splice sites. We used individual information scores in a forced mismatch analysis to examine local nucleotide changes that compensate for poor matches to consensus sequences. In addition, our analysis of distributions of individual information supported the model that the strength of each splice site depends upon the lengths of introns and exons in its neighborhood. Finally, we used the bootstrap method, a technique from computational statistics, to assess regional nucleotide content changes when the spliceosome machine is strained.

Previous studies have provided evidence suggesting that the spliceosome brings together pairs of exon or intron ends, a process referred to as exon and intron definition [19,24]. Our current work supports these models and suggests that a spliceosome complex might be even broader in its action, recruiting more than pairs of splice sites. We might consider the spliceosome as providing an interaction surface for recruiting sets of donors and acceptor sites in a process analogous to a purse string effect, with the exons and introns looping out away from the interaction surface. In this model, the interactions would be ordered (donor, acceptor, donor etc.), and interactions with the spliceosome would be aided by successful interactions of neighboring splice sites (indicated by the interdependence of neighborhood binding site strengths and their relationships to intron and exon lengths). The interactions would be polarized as suggested by the preference for C-rich pyrimidine tracts on the 3' side, and U-rich pyrimidine tracts on the 5' side, of longer introns. The interactions of short splice elements would be facilitated by good adherence to consensus sequences at D-1 to D6 and A-6 to A-1, minimal secondary structure (high A, U content), and the minimization or absence of pyrimidine tracts, which may reduce molecular crowding. The interactions of long splice elements would also be aided by similar strong consensus sequences, some minimization of secondary structure, and rich long pyrimidine tracts.

The notion that splice site interactions are aided by neighboring interactions, perhaps in a synergistic manner, leads to the prediction that blocks of ordered splice sites would bind to the spliceosome machine, and that mutation of individual splice sites could lead to exon or intron skipping, as experimentally observed [19]. Recent mass spectrometry and associated studies [25-29] suggest that the spliceosome is a complex macromolecular machine that is pre-assembled prior to binding of pre-mRNA, consistent with the model that it might present an ordered interaction surface for binding of groups of splice sites. The idea that multiple splice sites cooperate in binding to the spliceosome may account in part for the fairly low levels of information found near each individual splice site [30].

In addition, our analysis of exon and intron length distributions, and their relationships to information requirements, suggests that the spliceosome machine has a strong preference for the common intermediate-length splice elements, which have the least need for strong binding sites and, therefore, have relatively lower levels of individual information. It is only when the strained spliceosome machine is processing pre-mRNAs with less common long or very short splice elements that strong binding sites with high individual information are required.

## Materials and methods

### Scanning algorithm to identify splice sites

To determine the splice sites for a given cDNA transcript, we used the scanning algorithm outlined below with the transcript and the corresponding genomic DNA. The pseudocode for the algorithm is presented in Additional data file 1. The algorithm uses the following parameters to specify the degree to which it enforces matching between the cDNA transcript and the genomic DNA: *S *(*P*), number of bases in scanning (polymorphism) window; *s *(*p*), number of required matches in scanning (polymorphism) window; *cDNAtail*, minimum size of cDNA tail needed to search for a new exon; *polyAtail*, minimum percentage of A's in cDNA tail needed to predict a polyA tail.

The algorithm is designed so that it can either ignore any polymorphisms or take into account the following polymorphisms: substitution, insertion, or deletion of a single base (see Step 4 below).

#### Step 1: initialize windows

Define windows of size *S *at the 5' ends of the cDNA transcript and genomic DNA.

#### Step 2: find matching windows

Move the genomic window downstream to the first position where at least *s *bases match with the corresponding bases in the static cDNA window.

#### Step 3: find first mismatch after windows

Starting at the ends of the two windows, scan downstream one base at a time in both sequences until the first base mismatch is found. If the algorithm does not test for polymorphisms, go to Step 5; otherwise, go to Step 4.

#### Step 4

##### (a) Substitution

Define windows of size *P *in both the cDNA transcript and genomic DNA starting at the mismatched bases. If the two windows match in at least *p *bases, record a substitution polymorphism and go to Step 3; otherwise, if the algorithm tests for insertion or deletion polymorphisms, go to (b); otherwise, go to Step 5.

##### (b) Insertion/deletion

Move the cDNA window downstream by one base. If the cDNA and genomic windows match in at least *p *bases, record an insertion polymorphism and go to Step 3; otherwise, move the cDNA window upstream by one base and the genomic window downstream by one base. If the cDNA and genomic windows match in at least *p *bases, record a deletion polymorphism and go to Step 3; otherwise, go to Step 5.

(After step 4, the mismatch position on the genomic DNA is approximately the 3' splice site on the current exon.)

#### Step 5: test for final exon

If the region downstream of the cDNA window contains ≥ *cDNAtail *bases and <*polyAtail *As, go to Step 6; otherwise, terminate the algorithm. (This test is important to avoid identifying small, incorrect introns in Step 7.)

#### Step 6: find approximate position of 3' splice site

Define windows of size *S *starting at the current mismatched bases in the two sequences. Repeat Step 2 to determine a second downstream matching region on the genomic DNA - the intervening region is the predicted intron.

#### Step 7: find exact location of intron

If the first base upstream of the region and the last base in the region do not match, record their positions as the locations of the donor and acceptor splice sites and go to step 8. Otherwise, while the first base upstream of the region and the last base in the region match, perform the following consensus test: if the pattern GU..AG or AU..AC is found at the ends of the region, record the boundary positions as the locations of the donor and acceptor splice sites and go to step 8; otherwise, move the start and finish positions of the region one base upstream.

If a weak form of either pattern (three out of four bases matching) is found at the ends of the region, record the boundary positions as above; otherwise, terminate the algorithm.

#### Step 8: find first mismatch after windows

Repeat Step 3 using the second matching window of the genomic DNA and the corresponding cDNA window.

### Measuring individual information

We used individual information [6] over defined nucleotide intervals near splice sites to score how well individual instances of sequences matched the sequence conservation at splice sites. Suppose *S *is a set of *n *aligned sequences, each of length *m*, and *s*_1_, ..., *s*_m _denotes the nucleotides in a sequence *s *∈ *S*. Given a position 1 ≤ *p *≤ *m *and nucleotide α = A, C, G, or U, define the frequency of occurrence of α at position *p *by:

*f*_p_(α) = |{*s *∈ *S *| *s*_p _= α }|/*n *    (1)

The set of values 2 + *log*_2_(*f*_p_(α)) - γ, where 1 ≤ *p *≤ *m *and α = A, C, G, or U, defines the individual information weight matrix for *S*. The value γ is a correction factor for the sample size *n*, which has the approximate value 1.5/*ln*(2)*n *for *n *≥ 125 (see [4,31] for further discussion). The weight matrix is used to define the score of each sequence: given *s *∈ *S*, define;

*score*(*s*) = Σ{2 + *log*_2_(*f*_p_(*s*_p_)) - γ | 1 ≤ *p *≤ *m*}     (2)

This value is called the individual information of *s*. It is a real number with a maximum value of 2*m *that provides a quantitative assessment of how well *s *conforms to the conservation determined by the alignment. For example, *score*(*s*) < 0 indicates that *s *is a weak match to the consensus while *score*(*s*) > 0 indicates a better match to the consensus.

In [6], it is established that the average score for the set of sequences *S *equals the total information for the alignment:

Σ{*score*(*s*) | *s *∈ *S*}/*n *= *information*(1..*m*) = Σ{*information*(*p*) | 1 ≤ *p *≤ *m*}     (3)

where the *information *at position *p *is defined by:

*information*(*p*) = 2 - Σ{-*f*_p_(α) *log*_2_(*f*_p_(α)) | α = A, C, G, or U} - γ     (4)

Therefore, the distribution of scores for *S *has the average value *information*(1..*m*).

In certain cases, we want to assess how well a nucleotide sequence conforms to the consensus represented by *S *even if it is not a member. To define scores for arbitrary nucleotide sequences of length *m *that may contain at certain positions nucleotides not found in the original alignment, we modify (1) by using frequency pseudocounts in place of frequency counts:

*f*_p_*(α) = (|{*s *∈ *S *| *s*_p _= α}| + 1)/(*n *+ 4).     (1*)

This definition guarantees that for any sequence *s *of length *m*, *f*_p_*(α) > 0 for every nucleotide α and position *p*. The resulting individual information weight matrix is {2 + *log*_2_(*f*_p_*(α)) - γ} and we define the modified score of *s *by:

score*(*s*) = Σ{2 + *log*_2_(*f*_p_*(*s*_p_)) - γ | 1 ≤ *p *≤ *m*}     (2*)

The definition of individual information above (equation 2) assumes that each nucleotide has a background frequency of 0.25. Alternatively, one could use an individual 'relative entropy' measure such as:

*score*_b_(*s*) = Σ{*log*_2_(*f*_p_(α)/*b*_α_) - γ | α = *s*_p_, 1 ≤ *p *≤ *m*}     (2_b_)

that takes into account a set of background frequencies (*b*_α_).

However, choosing an appropriate set of source sequences to calculate background frequencies can be problematic and, for this reason, we used the scores defined by (2) for our analysis.

### Bootstrap method

The bootstrap method is described in [32]. We used this method to compare nucleotide contents in 32 nucleotide long windows adjacent to different groups of splice sites by testing the null hypothesis that the mean nucleotide contents in the windows (with respect to a given base) are equal for the different groups. The method was used in place of the two-sample *t* test [4] to avoid making any assumptions about the probability distributions of the groups including equal variances.

Each test was performed for a certain region associated with a splice element (for example, the nucleotide window -32..-1 at a donor site) and compared the mean nucleotide contents in the region for the different groups (for example, introns with length 64 to 1,023 versus introns with length 2,048 to 16,383). For the splice elements, we used three different types of groups classified by length: short, intermediate and long (see Figure 4 legend).

The following steps outline how the bootstrap method is used to test the equality of the means of two groups of nucleotide counts *G*_1 _and *G*_2_.

1. Compute the *t*'-statistic for *G*_1 _and *G*_2 _using the expression *t*' = (*m*_1 _- *m*_2_)/*s*, where *s*^2 ^= *s*_1_^2^/*n*_1 _+ *s*_2_^2^/*n*_2_, *m*_k _is the mean of *G*_k_, *n*_k _is the size of *G*_k_, and *s*_k_^2 ^is the variance of *G*_k_.

2. Normalize the values in each group *G*_k _by subtracting *m*_k _from every value in *G*_k_. Then the resulting mean of each group is zero.

3. For a fixed number of iterations *R*, perform the following steps:

(a) For each *k*, select a random bootstrap sample *B*_k _of size *n*_k _from *G*_k _with replacement (so the probability of choosing a given member of *G*_k _is always 1/*n*_k_).

(b) Compute and record the *t*'-statistic for the bootstrap samples *B*_1 _and *B*_2_.

For a probability threshold of α the original *t*'-statistic is significant at the 100α% level if *t*' lies among the 100α% largest bootstrap values recorded in Step 3(b). In this case, we reject the null hypothesis that the original means *m*_1 _and *m*_2 _are equal with Type I error α.

In our case, for each bootstrap test, we used a 1% significance level (α = 0.01) and 1,000 iterations (*R *= 1,000). The results of the bootstrap tests are summarized in Additional data file 2 and Figure 4.

## Additional data files

The following additional data are available with the online version of this paper. Additional data file 1 is the new algorithm for computing splice sites. Additional data file 2 provides results of the bootstrap tests. Additional data file 3 is a spread sheet with the full dataset of 24,423 predicted introns, which includes the nucleotide sequences (positions -32 to 32) flanking the donor and acceptor sites.

## Supplementary Material

Additional data file 1 Algorithm used for computing splice sites Click here for file

Additional data file 2 Results of the bootstrap tests Click here for file

Additional data file 3 The full dataset of 24,423 predicted introns, which includes the nucleotide sequences (positions -32 to 32) flanking the donor and acceptor sites Click here for file

## Figures and Tables

**Table 1:** Summary of introns and exons

	Parsed cDNAs*	Introns found	Exons found	Introns (length <20)	Exons (length <20)	Introns (length >8,191)	Exons (length >2,023)
No substitutions, no gaps^†^	5,092	14,559	19,474	63	1	311	418
Substitutions, no gaps^‡^	8,156	22,950	30,605	173	36	489	653
Substitutions, gaps^§^	8,234	24,753	32,987	40	38	576	761

*Number of cDNAs parsed successfully from a set of 10,057 cDNA transcripts. ^†^No single nucleotide substitutions, deletions or insertions allowed (S = 20; s = 20; P = 20, p = 20; see Materials and methods: scanning algorithm). ^‡^Single nucleotide substitutions allowed but deletions or insertions not allowed (S = 20; s = 18; P = 20, p = 18). ^§^Single nucleotide substitutions, insertions or deletions allowed (S = 20; s = 18; P = 20, p = 18).

**Table 2:** Forced mismatch analysis

Individual information	Number of matches to consensus at D-1 to D6 (introns 1,024 to 4,095)*					Total
		
	3-of-7	4-of-7	5-of-7	6-of-7	7-of-7	
<0	7 (7)^†^	41 (30)	2 (2)	0	0	50
0-6	0	37 (22)	334 (77)	26 (6)	0	397
6-11	0	0	26 (3)	948 (23)	0	974
>11	0	0	0	0	703 (2)	703
Total	7	78	362	974	703	2,124^‡^

*Number of matches to donor consensus GGU [A|G]AGU. ^†^Number of donor sites (number of different sequences). ^‡^The 2,124 donor sites do not include 3 sites with 2-of-7 matches and 1 site with 1-of-7 matches.
