# Supplementary material for: Challenging the spliceosome machine
Source: Genome Biol. 2006 Jan 17;7(1):R3. doi: 10.1186/gb-2006-7-1-r3 (PMC1431713; doi:10.1186/gb-2006-7-1-r3)
Supplement: Additional data file 1 — Algorithm used for computing splice sites [file gb-2006-7-1-r3-S1.doc]

# M. Weir, M. Eaton, M. Rice

# Supplementary Materials

**Scanning Algorithm to Identify Splice Sites**

To determine the splice sites for a given cDNA transcript, we used the following algorithm with the transcript and the corresponding genomic DNA. It uses the following parameters to specify the degree of matching between the cDNA and the genomic DNA:

*S* (*P*) -- number of bases in scanning (polymorphism) window

*s* (*p*) -- number of required matches in scanning (polymorphism) window

*cDNAtail* -- minimum size of cDNA tail needed to search for a new exon

*polyAtail* -- minimum fraction of A’s in cDNA needed to predict a polyA tail

*lengthcDNA* -- number of bases in cDNA transcript

The algorithm is designed so that it can either ignore any polymorphisms or test for the following polymorphisms: substitution, insertion, or deletion of a single base by specifying a true or false value for the following additional parameters:

*allowsubs* -- specifies if algorithm tests for substitution polymorphisms

*allowinsordel* -- specifies if algorithm tests for insertion or deletion polymorphisms

In the following pseudocode, the genomic DNA (cDNA transcript) is denoted by *dna* (*cdna*). The genomic window (cDNA window) from position *n* (*c*) to position *t* (*d*) is denoted by *dna*[*n***..***t*] (*cdna*[*c***..***d*]). The number of bases that match at the corresponding positions *n* and *c*, *n*+1 and *c*+1, … is denoted by | *dna*[*n***..***t*] ∩ *cdna*[*c***..***d*] |.

*c*, *m*, *processing* ← 1, 1, True

-- find first position *n* where genomic window starting at *n*

-- matches cDNA window starting at *c* in at least *s* bases

*n* ← *findMatchingWindows*(*S*, *s*, *c*, *m*)

**while** (*processing*)

{ -- find position *m* near 5' splice site of intron

*c*, *m* ← *extendMatchingWindows*(*P*, *p*, *allowinsert*, *c*, *n*)

-- test if final exon has been found based on short remaining cDNA tail or long polyA tail

**if** (*lengthcdna* – *c* < *cDNAtail*

**or** | *cdna*[*c* **..** *lengthcDNA*] ∩ 'A **..** A' | / (*lengthcdna* – *c*) ≥ *polyAtail*)

*processing* ← False

**else**

{ -- find position *n* near 3' splice site of intron

*n* ← *findMatchingWindows*(*S*, *s*, *c*, *m*)

-- find exact location of intron

*m*, *n*, *processing* ← *findIntron*(*m*, *n*)

}

}

The three procedures used in the description of the algorithm are specified below.

-- returns first position *n* ≥ *m* such that genomic window of length *S* starting at *n*

-- matches cDNA window of length *S* starting at *c* in at least *s* bases

1. *findMatchingWindows*(*S*, *s*, *c*, *m*)

{ *n* ← *m*

**while** (| *dna*[*n* **..** *n* + *S* - 1] ∩ *cdna*[*c* **..** *c* + *S* - 1] | < *s*)

*n* ← *n* + 1

**return**(*n*)

}

-- returns positions *c* and *m* of first mismatch following genomic DNA and cDNA windows

-- of length *P* that is not a polymorphism

2. *extendMatchingWindows*(*P*, *p*, *allowinsert*, *c*, *n*)

{ -- reset positions to end of windows

*j* ← *n* + *S* – 1

*c* ← *c* + *S* – 1

**while** (*True*)

{ -- find first mismatch after genomic window

choose *smallest* *m* > *j* • *dna*[*m*] ≠ *cdna*[*c* + *m* - *j*]

-- reset starting position of cDNA window

*c* ← *c* + *m* - *j*

-- test for polymorphisms

**if** (*allowsubs*)

{

**if**  (| *dna*[*m* **..** *m* + *P*-1] ∩ *cdna*[*c* **..** *c* + *P*-1] | ≥ *p*)

{ -- substitution polymorphism

*SubstitutePolyM* ← *SubstitutePolyM* ∪ {*m*}

*j* ← *m*

}

**else if** (*allowinsordel* **and** | *dna*[*m*+1 **..** *m* + *P*] ∩ *cdna*[*c* **..** *c* + *P*-1] | ≥ *p*)

{ -- insertion polymorphism

*InsertPolyM* ← *InsertPolyM* ∪ {*m*}

*j* ← *m*

}

**else if** (*allowinsordel* **and** | *dna*[*m* **..** *m* + *P*-1] ∩ *cdna*[*c* + 1 **..** *c* + *P*] | ≥ *p*)

{ -- deletion polymorphism

*DeletePolyM* ← *DeletePolyM* ∪ {*m*}

*j* ← *m*

}

**else**

**return**(*c*, *m*)

}

}

}

-- returns *m*, *n*, and *found* such that *dna*[*m* **..** *n*-1] is the predicted intron

-- in the genomic DNA if *found* = True

3. *findIntron*(*m*, *n*)

{

**if** (*dna*[*m*-1] ≠ *dna*[*n*-1])

*found* ← True

**else**

{

*found* ← False

**while** (**not**(*found*) **and** *dna*[*m*-1] = *dna*[*n*-1])

{ -- test for canonical consensus sequences

**if** (*dna*[*m* **..** *m*+1] = 'GT' **and** *dna*[*n*-2 **..** *n*-1] = 'AG') **or**

(*dna*[*m* **..** *m*+1] = 'AT' **and** *dna*[*n*-2 **..** *n*-1] = 'AC')

*found* ← True

**else**

**{** -- backtrack one position

*m* ← *m* - 1

*n* ← *n* - 1

}

}

-- test for 3 out of 4 match to canonical consensus sequences

**if** **not**(*found*) **and** ((| *dna*[*m* **..** *m*+1] ∩ 'GT' | + | *dna*[*n*-2 **..** *n*-1] ∩ 'AG' | ≥ 3) **or**

(| *dna*[*m* **..** *m*+1] ∩ 'AT' | + | *dna*[*n*-2 **..** *n*-1] ∩ 'AC' | ≥ 3))

*found* ← True

}

**return** (*m*, *n*, *found*)

}
