# Supplementary material for: Challenging the spliceosome machine
Source: Genome Biol. 2006 Jan 17;7(1):R3. doi: 10.1186/gb-2006-7-1-r3 (PMC1431713; doi:10.1186/gb-2006-7-1-r3)
Supplement: Additional data file 2 — Results of the bootstrap tests [file gb-2006-7-1-r3-S2.doc]

# TABLE S1. Nucleotide Content Differences (%)

Short introns (48-59) compared to intermediate introns (64-1023)

|  |  | D-32 to D-1 | D1 to D32 | A-32 to A-1 | A1 to A32 |
| --- | --- | --- | --- | --- | --- |
| Intron Before | A | +0.13 | **+0.94** | **+2.19** | +0.23 |
|  | C | **-1.01** | **-1.18** | **-2.15** | **-1.04** |
|  | G | **-0.58** | **-1.16** | **-0.51** | **-0.80** |
|  | U | **+1.46** | **+1.44** | +0.45 | **+1.58** |
| Varied Intron | A | **+0.71** | **+0.71** | **+2.47** | +0.27 |
|  | C | **-1.28** | **-1.28** | **-2.19** | **-1.24** |
|  | G | **-0.45** | **-0.45** | **+0.89** | **-0.43** |
|  | U | **+1.03** | **+1.03** | **-1.19** | **+1.36** |
| Intron After | A | **+0.87** | **+1.15** | **+1.46** | **+0.57** |
|  | C | **-1.32** | **-1.77** | **-1.65** | **-0.60** |
|  | G | **-0.92** | **-1.26** | **-0.54** | **-1.23** |
|  | U | **+1.34** | **+1.94** | **+0.73** | **+1.28** |

**Bold red**: short introns have significantly lower nucleotide content, p > 0.99 by a bootstrap test (see text); **Bold green**: short introns, significantly higher, p > 0.99

Long introns (2048-16383) compared to intermediate introns (64-1023)

|  |  | D-32 to D-1 | D1 to D32 | A-32 to A-1 | A1 to A32 |
| --- | --- | --- | --- | --- | --- |
| Intron Before | A | **+1.30** | **+0.88** | **-1.25** | **+2.30** |
|  | C | **-0.81** | +0.10 | +0.07 | -0.31 |
|  | G | -0.64 | -0.43 | **-0.76** | **-1.49** |
|  | U | +0.13 | -0.46 | **+2.14** | -0.48 |
| Varied Intron | A | **+2.33** | +0.41 | **-3.36** | **+1.30** |
|  | C | **-0.94** | +0.32 | **+1.11** | +0.01 |
|  | G | **-0.77** | -0.20 | -0.31 | **-0.54** |
|  | U | **-0.56** | -0.55 | **+2.56** | **-0.78** |
| Intron After | A | +0.02 | **+1.12** | **-1.22** | +0.06 |
|  | C | +0.30 | -0.27 | **+0.99** | **+0.83** |
|  | G | +0.30 | +0.11 | -0.28 | -0.05 |
|  | U | **-0.69** | **-0.89** | +0.50 | **-0.87** |

**Bold red**: long introns have significantly lower nucleotide content, p > 0.99 by a bootstrap test (see text); **Bold green**: long introns, significantly higher, p > 0.99

Short exons (32-90) compared to intermediate exons (128-511)

|  |  | D-32 to D-1 | D1 to D32 | A-32 to A-1 | A1 to A32 |
| --- | --- | --- | --- | --- | --- |
| Intron Before | A | **+0.76** | +0.44 | -0.29 | **+2.02** |
|  | C | -0.48 | +0.27 | **-0.78** | **-1.92** |
|  | G | -0.52 | +0.14 | -0.12 | -0.23 |
|  | U | +0.23 | **-0.89** | **+1.34** | +0.19 |
| Intron After | A | **+2.00** | **+1.09** | -0.05 | **+0.94** |
|  | C | **-1.83** | -0.33 | +0.11 | +0.16 |
|  | G | **-2.35** | **-1.02** | **+0.36** | **-0.70** |
|  | U | **+2.24** | +0.28 | -0.41 | -0.36 |

**Bold red**: short exons have significantly lower nucleotide content, p > 0.99 by a bootstrap test (see text); **Bold green**: short exons, significantly higher, p > 0.99

Long exons (1048-4095) compared to intermediate exons (128-511)

|  |  | D-32 to D-1 | D1 to D32 | A-32 to A-1 | A1 to A32 |
| --- | --- | --- | --- | --- | --- |
| Intron Before | A | **+0.99** | **+0.80** | +0.27 | +0.31 |
|  | C | -0.27 | -0.47 | +0.36 | **+0.74** |
|  | G | +0.16 | +0.09 | **-0.58** | +0.02 |
|  | U | **-0.87** | -0.41 | -0.05 | **-1.07** |
| Intron After | A | +0.48 | +0.50 | +0.13 | +0.53 |
|  | C | +0.18 | **-2.35** | **-1.53** | +0.24 |
|  | G | +0.44 | **-1.38** | **-1.04** | **-0.62** |
|  | U | **-1.03** | **+3.20** | **+2.40** | -0.14 |

**Bold red**: long exons have significantly lower nucleotide content, p > 0.99 by a bootstrap test (see text); **Bold green**: long exons, significantly higher, p > 0.99
